# Clinical and prognostic insights into radiation-associated angiosarcoma: a multi-institutional analysis

**DOI:** 10.1186/s13014-026-02895-w

**Published:** 2026-08-19

**Authors:** Melissa Harbrücker, Sebastian Hoffmann, Johannes Tobias Thiel, Noelle Samira Jacob-Rehfeld, Sebastian M. Christ, Adrien Daigeler, Anna Duprée, Felix Ehret, Anne Flörcken, Peter Hohenberger, Bernd Kasper, Christoph Reißfelder, Jens Jakob, Ulrich Kneser, Felix Strübing, Jana Käthe Striefler, Siyer Roohani

**Affiliations:** 1 Surgical Clinic, Faculty of Medicine Mannheim, University Medicine Mannheim, Heidelberg University, Theodor-Kutzer-Ufer 1-3, 68167 Mannheim, Germany; 2 DKFZ Hector Cancer Institute at the University Medical Center Mannheim, Mannheim, Germany; 3 Department of Hand, Plastic, Reconstructive and Burn Surgery, BG Trauma Center Tübingen, University of Tübingen, Schnarrenbergstraße 95, 72076 Tübingen, Germany; 4 Department of Internal Medicine II, Oncology/Hematology/BMT/Pneumology, University Medical Center Hamburg-Eppendorf, Hamburg, Germany; 5 Department of Radiation Oncology, University Hospital Zurich, Zurich, Switzerland; 6 Charité - Universitätsmedizin Berlin, Corporate Member of Freie Universität Berlin and Humboldt-Universität zu Berlin, Department of Radiation Oncology, Augustenburger Platz 1, 13353 Berlin, Germany; 7 Department of General, Visceral, and Thoracic Surgery, University Medical Center Hamburg-Eppendorf, Hamburg, Germany; 8 Charité - Universitätsmedizin Berlin, Corporate Member of Freie Universität Berlin and Humboldt-Universität zu Berlin, Department of Hematology, Oncology, and Tumor Immunology, Berlin, Germany; 9 German Cancer Consortium (DKTK), Partner Site Berlin, a Partnership Between DKFZ and Charité-Universitätsmedizin Berlin, Berlin, Germany; 10 Division of Surgical Oncology and Thoracic Surgery, University Medical Center Mannheim, Medical Faculty Mannheim - Heidelberg University, Heidelberg, Germany; 11 Sarcoma Unit, University of Heidelberg, Mannheim University Medical Center, Mannheim Cancer Center (MCC), Mannheim, Germany; 12 Sarcoma Unit, Department of Surgery, University Medical Center and Medical Faculty Mannheim, University of Heidelberg, Mannheim, Germany; 13 Department of Hand, Plastic and Reconstructive Surgery, Microsurgery, Burn Trauma Center, BG Trauma Center Ludwigshafen, University of Heidelberg, 67071 Ludwigshafen, Germany; 14 Berlin Institute of Health at Charité - Universitätsmedizin Berlin, BIH Biomedical Innovation Academy, BIH Charité (Junior) Clinician Scientist Program, Berlin, Germany

**Keywords:** Soft tissue sarcoma, Breast cancer, Secondary malignancy, Toxicity, Cancer survivorship

## Abstract

**Purpose:**

To evaluate oncological outcomes and prognostic factors in patients with radiation-associated angiosarcoma (RAAS) treated at referral centers.

**Methods:**

We conducted a multi-institutional retrospective cohort study of patients with histopathologically confirmed primary or recurrent RAAS treated at four referral centers in Germany. Endpoints were overall survival (OS) and progression-free survival (PFS), estimated using the Kaplan–Meier method. Prognostic factors were assessed using multivariable Cox regression.

**Results:**

Among 71 patients (97.2% female; median age 69 years), 90% had a history of breast cancer treated with radiotherapy (RT). RAAS developed after a mean latency of 7 years, with a median RT dose of 59.4 Gy. Over a median follow-up of 13.0 months, OS reached a median of 41.7 months, with 1- and 2-year rates of 82.9% and 61.7%, respectively. Median PFS was 9.5 months, with 1- and 2-year rates of 43.7% and 27.1%. Larger tumor size (HR 1.09, *p* = 0.032) and metastatic disease at diagnosis (HR 2.98, *p* = 0.003) were associated with worse OS.

**Conclusions:**

RAAS are aggressive, frequently relapsing malignancies occurring years after RT. Larger tumor size and metastatic presentation are associated with worse OS. Multi-institutional and translational studies are needed to clarify disease biology, refine risk factors, and guide treatment strategies.

## Introduction

Radiation-associated sarcomas (RAS) are classified as ultra-rare sarcomas, with an estimated incidence rate between 0.05 and 0.3 per 100,000 individuals [1, 2]. These tumors represent a severe but infrequent complication of radiotherapy, arising in the context of a previously treated primary malignancy. Despite their rarity, RAS warrant significant clinical attention due to their aggressive nature and poor prognosis [3–5]. Previous cohort studies have demonstrated that RAS predominantly affect women who have undergone radiotherapy for breast cancer, with radiation-associated angiosarcoma (RAAS) being the most common histologic subtype [6–8]. RAAS typically develop several years after the initial radiotherapy, with a latency period of approximately 6–7 years [9]. The diagnosis is often challenging due to the variable and sometimes subtle clinical presentation. Most RAAS cases are diagnosed at an advanced stage, with metastases already present at the time of diagnosis. The standard treatment for RAAS is surgical resection [3]; however, achieving clear margins is frequently difficult because of the tumor’s diffuse growth pattern [10]. Reirradiation remains a therapeutic option. Systemic therapies have also been explored, and anthracycline and taxanes have been established as systemic treatment options [11]. Despite these efforts, the prognosis for RAAS remains poor, with 5-year overall survival (OS) rates of 27–48% [12]. However, in selected patients with radiation-associated breast angiosarcoma, radical surgical resection has been associated with substantially improved outcomes, including 5-year disease-specific survival rates of up to 86% [13]. Identifying risk factors beyond radiotherapy is critical for improving prevention and early detection strategies. Chronic lymphedema and genetic predispositions are among the primary risk factors [14]. However, many aspects of the pathogenesis and prognostic factors remain poorly understood. This multi-institutional study aims to provide a comprehensive analysis of the clinical and prognostic risk factors associated with RAAS, offering new insights into this rare and aggressive complication of cancer treatment.

## Materials and methods

This retrospective, multi-institutional cohort study included patients with histopathological confirmed diagnoses of RAAS who underwent treatment at four sarcoma referral centers in Germany between 2004 and 2023. The four participating German centers were Charité – Universitätsmedizin Berlin, University Medical Center Hamburg-Eppendorf (UKE), University Medical Center Mannheim, and BG Trauma Center Tübingen. Data were reviewed on patient demographics, imaging findings, pathology reports, surgical interventions, oncological treatments, including radiotherapy and chemotherapy, and oncological outcomes. Data on the extent of surgical resection were collected where available. Given the retrospective design and long study period, detailed operative reports were not consistently accessible for all patients. However, all procedures were performed at specialized sarcoma centers with the intent of achieving wide excision of all clinically involved and suspected infiltrated tissue. All cases were reviewed by dedicated musculoskeletal pathologists and radiologists. The study endpoints comprised OS and progression-free survival (PFS). OS was defined as the interval from initial treatment to death from any cause. PFS was defined as the interval from initial treatment to the detection of disease progression at any site, as determined by histopathological or radiographic evidence on follow-up imaging (mammography, ultrasound, computed tomography or magnetic resonance imaging [MRI]) or by biopsy or death by any cause. Clinical follow-up was calculated from the date of initial treatment to the date of the last clinical visit. Patients were censored at the time of their last available follow-up if no disease progression or death was observed. Survival status data were obtained from the tumor registries of the participating institutions. For descriptive statistics, ranges, means, medians, and interquartile ranges for continuous variables were used. OS and PFS were assessed using the Kaplan-Meier estimator. Multivariable Cox regression was performed to analyze the influence of clinical risk factors (tumor size, presentation status) as well as treatment modalities on OS and PFS. A p-value of ≤ 0.05 was considered statistically significant. The proportional hazards assumption was tested with Schoenfeld residuals. Statistical analysis and figures were done using STATA MP 16.0 (StataCorp, College Station, TX, USA). The study received institutional review board approval (EA1/072/23).

## Results

### Clinicopathologic and treatment characteristics

The clinicopathologic and treatment characteristics are summarized in Table 1. A total of 71 patients were included. Median age at diagnosis was 69 years (range: 36–87), and most patients (69, 97.2%) were female. Most RAAS were detected in the breast (60 of 71, 84.5%), with 6 cases (8.5%) arising in the chest wall and 5 cases (7%) occurring in other anatomical locations. Approximately half of the patients (34, 47.9%) presented with primary localized disease, while 23 cases (32.4%) exhibited localized, but recurrent disease after surgery elsewhere, 9 cases (12.7%) had metastatic disease, and 5 patients (7%) presented with metastatic recurrent disease after first-line treatment elsewhere.

The median tumor size was 4.9 cm (range: 1.2–22.4 cm). Among tumors with available size data, 31 (43.7%) measured ≤ 5 cm, while 25 (35.2%) measured > 5 cm. The primary malignancy treated with radiation prior to the development of RAAS was breast cancer in 63 patients (90%), while 3 patients (4.3%) had other malignancies. In 4 patients (5.7%), the primary tumor entity was not documented; however, prior radiotherapy, including the treated region and dose, was confirmed in all cases. The median interval between prior radiation therapy and the diagnosis of RAAS was 7 years. The median total radiation dose received prior to the RAAS diagnosis was 59.4 Gy.

**Fig. 1:**
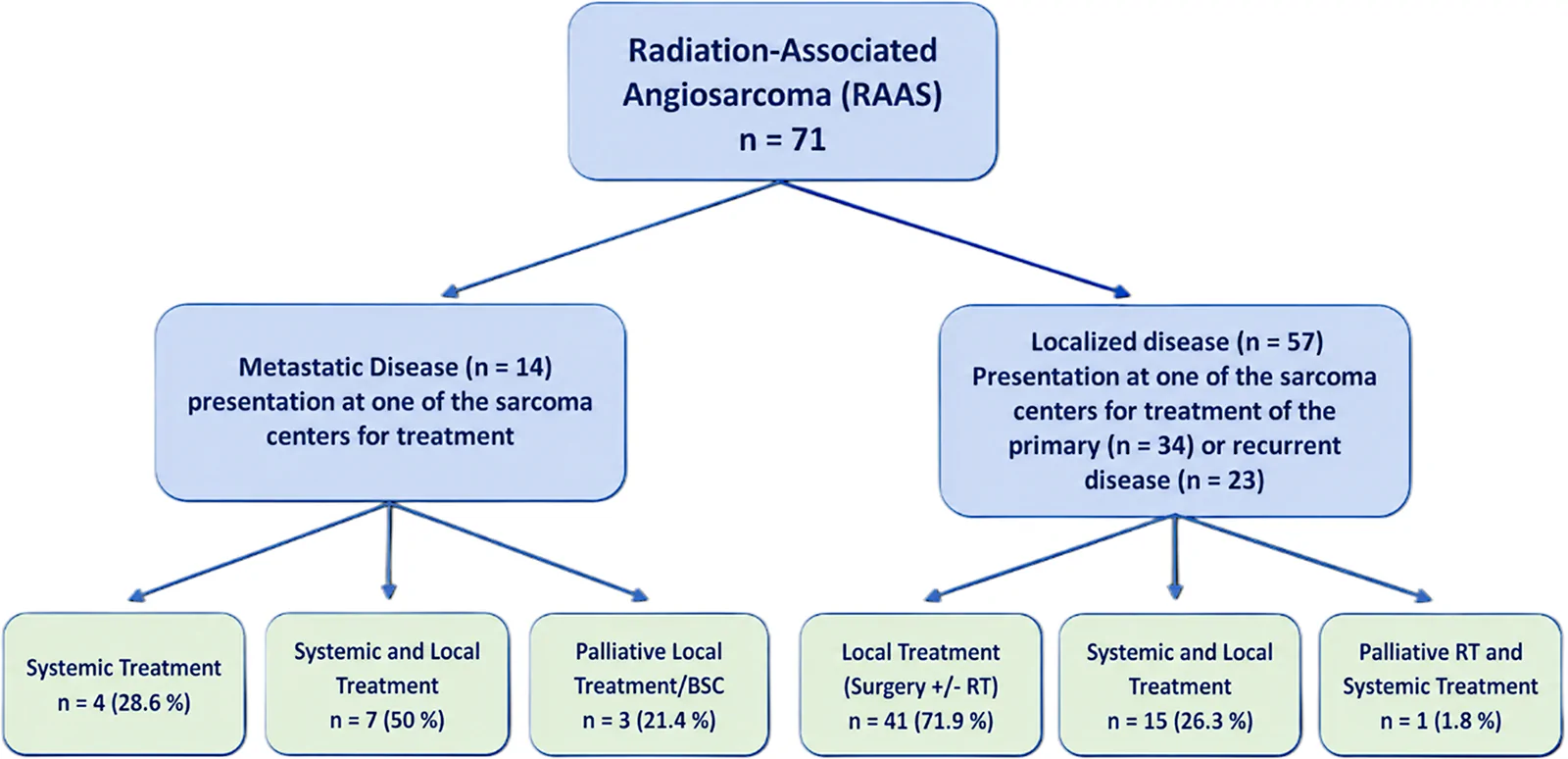
Treatment pathways of radiation-associated angiosarcoma (RAAS) stratified by presentation status

Surgical intervention was performed in 58 patients (81.7%), achieving clear margins (R0) in 47 cases (66.2%), microscopically positive margins (R1) in 4 cases (5.7%); information was unavailable for 7 patients (9.9%). Among 58 patients undergoing surgery, wide local excision was the most common approach (67.2%), while mastectomy and wide compartmental resection were each performed in 1 case (1.7%); information on surgical extent was not available in 17 cases (29.4%). A total of 10 patients (14.1%) received radiotherapy, most commonly in the preoperative setting (7%), with a median dose per fraction of 1.8 Gy and a median total dose of 50 Gy. Chemotherapy was administered to 25 patients (35.2%), predominantly postoperatively in 10 cases (14.1%). Among the 58 patients who underwent surgery, 36 patients (50.8%) were treated with surgery alone, 22 patients (31%) received surgery with perioperative radiotherapy or chemotherapy, and 2 patients (2.8%) received sequential radiotherapy and chemotherapy. Three patients received best supportive care only. An overview of treatment modalities divided by initial presentation status is depicted in Fig. 1.

**Table 1:** Clinicopathologic and treatment characteristics

	All *N* = 71		
Characteristics	*N*		%
Median age in years (range)	69 (36–87)		
Sex			
• Female	69	97.2	
• Male	2	2.8	
Site			
• Breast left	27	38	
• Breast right	24	33.8	
• Breast (side not specified)	9	12.7	
• Chest wall	6	8.5	
• Other*	5	7	
Presentation status			
• Localized, primary diagnosis	34	47.9	
• Localized, recurrent disease	23	32.4	
• Metastatic, primary diagnosis	9	12.7	
• Metastatic, recurrent disease	5	7	
Tumor size [cm]			
• Mean	6.4		
• Median	4.9		
• IQR	3-9.1		
• Range	1.2–22.4		
• ≤ 5 cm	31	43.7	
• > 5 cm	25	35.2	
• Information N/A	15	21.1	
Prior oncological history			
Primary tumor			
• Breast cancer	63	90	
• Others^#^	3	4.3	
• Information N/A	4	5.7	
Time in years between radiation and primary diagnosis of radiation-associated angiosarcoma			
• Mean	7.5		
• Median	7		
• IQR	5-8.8		
• Range	2–17		
• Information N/A	29	40.8	
Total radiation dose [Gy]			
• Mean	56.9		
• Median	59.4		
• IQR	52.6–60.4		
• Range	42.2–66.4		
• Information N/A	34	47.9	
Management of radiation-associated angiosarcoma			
Surgery			
• Yes	58	81.7	
• No	13	18.3	
Extent of surgical resection	58	100	
• Wide local excision	39	67.2	
• Mastectomy	1	1.7	
• Wide compartmental resection	1	1.7	
• Information N/A	17	29.4	
Tumor margin status			
• Negative (R0)	47	66.2	
• Positive microscopically (R1)	4	5.7	
• Information N/A	7	9.9	
Radiotherapy			
• Preoperative RT	5	7	
• Postoperative RT	3	4.2	
• Sequential RT and CTX	2	1.4	
• Median dose per fraction [Gy] (range)	1.8 (1.8-3)		
• Median total dose [Gy] (range)	50 (30–54)		
Chemotherapy			
• Preoperative CTX	4	5.6	
• Postoperative CTX	10	14.1	
• Pre- and postoperative CTX	1	1.4	
• CTX only	8	11.3	
• Sequential RT and CTX	2	1.4	
Combination therapies			
• Surgery only	36	50.8	
• Surgery with perioperative RT and/or CTX	22	31	
• RT and CTX	2	2.8	

^*^Other sites include extremities, bones, mediastinum, kidneys. ^#^Other primaries include lymphoma, chronic myelogenous leukemia

Abbreviations: CTX = Chemotherapy; Gy = Gray; N/A = Not available; RT = Radiotherapy

### Oncological outcomes

**Fig. 2:**
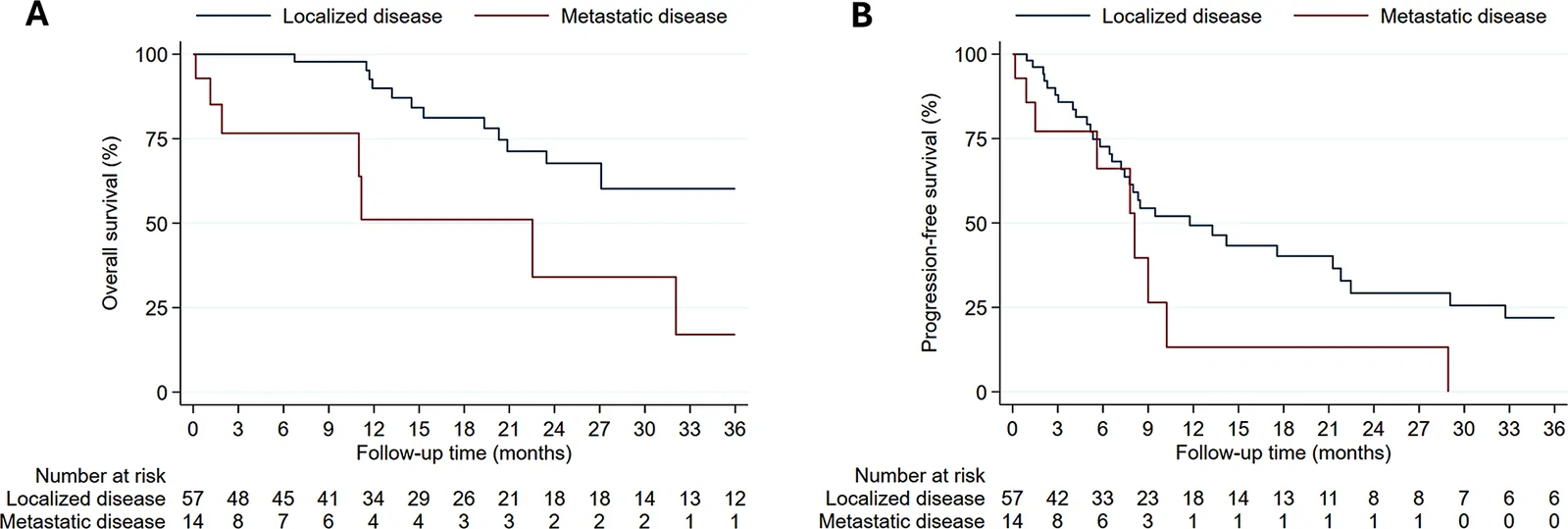
Overall survival (**A**) and progression-free survival (**B**) stratified by disease status at presentation

Oncological outcomes are detailed in Table 2. The median clinical follow-up was 13 months. During follow-up, 26 patients (36.6%) died, 30 (42.3%) experienced local recurrence, and 18 (25.4%) experienced distant disease progression. Outcomes differed markedly by disease at presentation. Among patients with localized disease (*n* = 57), median OS was 70.4 months, with 1- and 2-year OS rates of 89.9% and 67.7%, respectively (Fig. 2A). Median PFS was 11.8 months, with 1- and 2-year PFS rates of 49.3% and 29.2% (Fig. 2B). In contrast, patients presenting with metastatic disease (*n* = 14) had a median OS of 22.5 months, with 1- and 2-year OS rates of 51.1% and 34.1% (Fig. 2A). Median PFS was 8.1 months, with 1- and 2-year PFS rates of 13.2% and 13.2%, respectively (Fig. 2B).

**Table 2:** Oncological outcomes

	Median		Mean		IQR		Range	
**Clinical follow-up, months**	13.0		22.1		24.2		0.2-116.7	
Localized disease at presentation	*N* = 57							
	**Median** **(months)**	**3 months (%)**		**6 months (%)**		**12 months (%)**		**24 months (%)**
Overall survival	70.4 (CI: 23.5-not reached)	100		100		89.9 (CI: 75.2–96.1)		67.7 (CI: 48.9–81.0)
Progression-free survival	11.8 (CI: 7.4–21.8)	87.9 (CI: 75.0-94.4)		72.6 (CI: 57.5–83.1)		49.3 (CI: 33.9–63.0)		29.2 (CI: 15.2–44.8)
Metastatic disease at presentation	*N* = 14							
	**Median** **(months)**	**3 months (%)**		**6 months (%)**		**12 months (%)**		**24 months (%)**
Overall survival	22.5 (CI: 6.9-not reached)	76.6 (CI: 43.3–91.9)		76.6 (CI: 43.3–91.9)		51.1 (CI:17.4–77.2)		34.1 (CI:6.1–66.0)
Progression-free survival	8.1 (CI:1.5–10.2)	77.1 (CI: 44.2–92.1)		66.1 (CI: 31.6–86.2)		13.2 (0.7–43.6)		13.2 (0.7–43.6)

Abbreviations: CI = 95% confidence interval; IQR= Interquartile range

### Multivariable Cox regression analysis

On multivariable analysis, larger tumor size (HR 1.09, *p* = 0.032) and metastatic presentation at diagnosis (HR 2.98, *p* = 0.003) were significantly associated with worse OS. For PFS, treatment with chemotherapy with or without radiotherapy (HR 2.73, *p* = 0.045) was associated with significantly higher risk of PFS events compared to surgery alone (Table 3).

**Table 3:** Multivariable Cox proportional hazards model for overall survival and progression-free survival

Overall survival			
Variable	**Hazard ratio**	**Confidence interval (95%)**	**p-value**
Age (in years)	0.97	0.93–1.01	0.158
Tumor size (in cm)	1.09	1.01–1.18	0.032
Presentation status			
Localized disease	Reference		
Metastatic disease	2.98	1.88–21.1	0.003
Progression-free survival			
Age (in years)	0.98	0.95–1.02	0.266
Tumor size (in cm)	1.03	0.96–1.11	0.371
Therapy			
Surgery only	Reference		
Surgery + RT and/or CTX	1.41	0.59–3.38	0.445
CTX +/- RT	2.73	1.02–7.28	0.045

Abbreviations: CTX = Chemotherapy; RT = Radiotherapy

## Discussion

RAAS is a rare, aggressive malignancy that continues to pose significant diagnostic and therapeutic challenges. Its rarity has historically limited the evidence base to case reports and small series, making large-scale cohort analyses, such as the present study, critically important. This multi-institutional study describes one of the largest cohort analyses focusing on the prognostic factors for survival of RAAS patients. In line with the current literature, a late onset of RAAS was observed in our study, with a median onset of 7 years after radiotherapy [9, 15].

Tumor size per cm and metastatic disease status at presentation emerged as significant prognostic factors for OS in multivariable analysis. For cm of increase in tumor size was significantly associated with worse OS (*p* = 0.032), as did metastatic disease at presentation (*p* = 0.03). These findings reinforce the aggressive biological nature of RAAS and the importance of early detection. Given the rapid tumor growth typically associated with RAAS, prompt recognition and diagnosis are essential. It also highlights the diagnostic complexity of RAAS, which is often poorly demarcated and characterized by satellite lesions and morphological heterogeneity [16, 17]. Previous reviews have also identified tumor size to be associated with local recurrence-free survival or disease-free survival [18]. In our cohort, tumor size was not significantly associated with PFS. This discrepancy may be due to sampling bias, as the presentation at the center was usually not the presentation as a primary tumor and is therefore associated with a poorer prognosis. One important aspect to consider when interpreting our results is the heterogeneity of disease status at presentation. Our cohort includes both patients with locally advanced and metastatic disease, which may limit the direct comparability of oncological outcomes. However, given the rarity of radiation-associated angiosarcoma, further stratification would have substantially reduced sample size and limited the robustness of subgroup analyses. Moreover, this heterogeneity reflects real-world clinical practice, where patients frequently present across a spectrum of disease stages.

Early presentation at a center is crucial and can have a positive impact on the outcome of sarcoma patients [19]. UK guidelines emphasize that referral to specialized sarcoma centers is essential, as management within expert multidisciplinary tumor boards is associated with improved outcomes [20]. This is also supported by a recent French study demonstrating better survival for sarcoma patients treated in specialist centers [21]. Furthermore, an audit of the Scottish NHS has resulted in a clear recommendation to treat breast sarcoma cases at specialized sarcoma units [22]. In our cohort, only about 40% were confirmed to be initially treated at a certified center. This finding underscores the necessity for appropriate postoperative and, more importantly, long-term surveillance for secondary malignancies in breast cancer patients who have undergone radiotherapy. Currently, German breast cancer guidelines recommend imaging follow-up, such as X-ray or MRI, only in high-risk patients, with annual follow-up intervals after the sixth year. Our findings advocate for more nuanced guidelines. We propose a structured RAAS screening program for patients who have received radiotherapy, beginning 6–8 years post-treatment, incorporating MRI for early detection of vascular anomalies. Sufficient cooperation between breast and sarcoma centers is the basic prerequisite for this.

Surgery remains the cornerstone of RAAS management, performed in 81.7% of patients in our cohort. Despite extensive debate on optimal resection strategies, including margin width and resection of the complete irradiated tissue fields, our analysis found no significant association between margin status and PFS. The strategies of the resections cannot be optimally evaluated retrospectively in the present cohort. Nevertheless, surgical resection with 2–3 cm safety margins and subsequent plastic reconstruction remains a widely accepted approach [13, 23–25]. Although pathological margin status remains the current standard for postoperative assessment, its prognostic value in RAAS may be limited due to the infiltrative and multifocal growth pattern of the disease, which challenges both surgical delineation and histopathological evaluation. However, the level of evidence supporting these margin recommendations is low, and the appropriate metric distances remain debatable. While surgery remains a key aspect of RAAS management, the optimal extent of resection is still under debate, and there is currently no consensus on whether resection should be limited to visible disease or extended to the entire region of the irradiated field, particularly given the lack of standardized integration of prior radiotherapy plans and radiation isodoses into surgical decision-making. In addition, the frequent multifocal growth pattern of radiation-associated sarcomas cells into question the reliability of negative margin assessment in this context. Harati et al. identified negative surgical margins as the sole positive prognostic factor for OS [25]. However, their study cohort was not exclusively composed of RAAS cases. Li et al. reported in a large cohort of 76 women, that in patients with radiation-associated breast angiosarcoma, radical resection of the entire irradiated skin field significantly reduced recurrence and improved survival, achieving a 5-year DSS of 86% [13]. While negative margins remained prognostic, the authors emphasized that multifocality limits the eliability of margin assessment [13]. Whether complete resection of the radiation field provides additional benefit remains unclear and warrants further prospective study. Moreover, it appears that good results from radical surgery can only be achieved as part of a multimodal approach.

Recent studies, including the Dutch cohort by van der Burg et al., showed survival benefits of multimodal therapy with neoadjuvant taxanes in breast cancer-associated RAAS [26, 27]. It was demonstrated that 3-year local recurrence-free survival, distant metastasis-free survival, and OS could be significantly improved by neoadjuvant taxane therapy in patients with RAAS after breast cancer treatment [27]. In our cohort, multimodal therapy did not demonstrate a clear advantage over surgery alone, most likely reflecting delayed diagnosis, initial treatment outside certified centers, and the heterogeneity of systemic regimens across the long study period. The heterogeneity of multimodal approaches, including varying neoadjuvant and adjuvant strategies influenced by prior therapies, limits comparability between specific regimens; therefore, our analysis focused on broader treatment categories rather than individual protocols. While other studies on neoadjuvant chemotherapy in angiosarcoma suggest a potential benefit, consistent improvements in oncological outcomes have not been conclusively demonstrated [28–30].

RAAS also appear responsive to immunotherapy, which may further improve prognosis despite the absence of predictive biomarkers [31]. Prospective data, such as the trial by Pink et al. with paclitaxel and pazopanib, underline the potential of modern systemic combination therapies [32]. To date, no reliable biomarker exists to predict immunotherapy response in RAAS. While c-myc has been investigated as a potential therapeutic target, its clinical application remains limited to singular studies [33, 34]. Large-scale cohorts such as the Canadian Sarcoma Research and Clinical Collaboration (CanSarCC) have contributed valuable insights, though without yielding actionable therapies [23]. Notably, the “Count Me In” project evaluated ipilimumab and nivolumab in cutaneous angiosarcoma with promising early results, illustrating the potential of patient-driven, collaborative research efforts [35].

At present, available albeit limited data suggest that reirradation for RAAS can achieve high rates of local disease control with moderate toxicity and is recommended after individualized consideration at expert sarcoma centers [36]. Across published studies, either hyperfractionated or conventionally fractionated regimens in combination with chemotherapy or regional hyperthermia were employed, with the aim of minimizing radiation exposure to previously irradiated tissues while maintaining antitumor efficacy. In a small patient cohort, preoperative hyperfractionated reirradiation demonstrated high local control but was associated with considerable acute toxicity [37]. Similarly, perioperative hyperfractionated proton reirradiation achieved no local recurrences at a median follow-up of 1.5 years, with only moderate toxicity observed [38]. The use of preoperative taxane-based chemotherapy in combination with radiotherapy for breast RAAS has also shown promising outcomes, with notable pathological responses and effective local and distant disease control, albeit at the cost of wound-related toxicity and a frequent need for reconstructive procedures to achieve closure [39]. Previous retrospective analyses suggest that preoperative reirradiation combined with regional hyperthermia and preoperative chemotherapy may yield favorable outcomes over no preoperative chemotherapy [27]. Finally, conventionally fractionated reirradiation with reduced doses, when paired with hyperthermia, provided considerable disease control, though some higher-grade toxicities were also reported [40]. In our cohort, photon reirradiation with a median dose of 50 Gy was administered. However, due to the considerable heterogeneity of the patient population and the absence of toxicity data, no definitive conclusions can be drawn. For clinical practice, it may therefore be concluded that careful evaluation of prior radiation plans, expected and existing toxicity, and patient preferences is essential. Strategies to minimize dose to previously irradiated tissues – such as proton therapy, hyperfractionation, or the addition of chemotherapy or regional hyperthermia – appear advisable. Ultimately, treatment decisions should be individualized and made within a multidisciplinary sarcoma expert team setting.

In the future, the DKFZ-Hector-funded ‘TETRIS’ initiative exemplifies a translational bench-to-bedside approach to identify molecular targets in radiation-associated sarcomas. Such initiatives highlight the urgent need for cross-disciplinary collaboration among breast cancer specialists, radiation oncologists, and sarcoma experts. Raising awareness, strengthening follow-up protocols, expanding expert training, and supporting registry-based research will be key to advancing care. Looking ahead, progress in molecular diagnostics, imaging, and targeted systemic therapies is likely to shape the future of RAAS management. Integration of next-generation sequencing and transcriptomic profiling may uncover actionable mutations or molecular signatures, while artificial intelligence-based imaging, particularly deep learning applied to breast MRI, could enable earlier detection of subtle vascular changes in early-stage RAAS [41]. Finally, structured treatment pathways and early referral to specialized sarcoma centers will help improve outcomes, underscoring the potential advantages of centralized and interdisciplinary care.

## Limitations

This study is subject to the limitations of a retrospective, multi-institutional design. Treatment strategies evolved over the long inclusion period (2004–2023), introducing variability in clinical management. Another important limitation is the heterogeneity of surgical strategies across centers, as detailed information on the extent of resection was not uniformly available. Comprehensive data on systemic therapy regimens and responses were often incomplete, limiting interpretation of treatment efficacy. Furthermore, radiotherapy plan data were unavailable, precluding a detailed analysis of dose–response relationships and potential dose–volume effects in the development of RAAS. A notable proportion of patients were initially managed outside of specialized sarcoma centers, which may have affected diagnostic accuracy, surgical quality, and overall care. Finally, although this represents one of the larger cohorts of radiation-associated angiosarcoma to date, the rarity of the disease constrains statistical power for subgroup analyses and limits the generalizability of the findings.

## Conclusion

Radiation-associated angiosarcoma remains a rare and aggressive malignancy with poor outcomes, particularly when not managed within specialized sarcoma centers. Our findings highlight substantial heterogeneity in diagnostic and therapeutic approaches across institutions, underscoring the need for greater uniformity in treatment strategies, including surgical management and multimodal concepts. Given the complexity of this disease and the limitations of current evidence, centralized care within expert multidisciplinary sarcoma teams appears essential to optimize outcomes. Furthermore, our results emphasize the urgent need for prospective, multicenter studies and registry-based approaches to better define standardized treatment pathways and improve patient prognosis.

## Data Availability

Data available on request from the corresponding author.

## Abbreviations

CT: Computed tomography
CTX: Chemotherapy
FNCLCC: Fédération Nationale des Centres de Lutte Contre le Cancer
IQR: Interquartile range
LC: Local control
MRI: Magnetic resonance imaging
N/A: Not available
OS: Overall survival
PFS: Progression-free survival
RAAS: Radiation-associated angiosarcoma
RAS: Radiation-associated sarcoma
RT: Radiotherapy
